# Lung function and paper dust exposure among workers in a soft tissue paper mill

**DOI:** 10.1007/s00420-019-01469-6

**Published:** 2019-08-26

**Authors:** Eva Andersson, Gerd Sällsten, Susanna Lohman, Richard Neitzel, Kjell Torén

**Affiliations:** 1 Occupational and Environmental Medicine, Sahlgrenska Academy and University Hospital, Box 414, 405 30 Göteborg, Sweden; 2 Department of Environmental Health Sciences, University of Michigan, Ann Arbor, MI USA

**Keywords:** Obstructive lung disease, Occupational, COPD, Organic dust

## Abstract

**Purpose:**

To study respiratory effects of exposure to soft paper dust exposure, a relationship that is rarely studied.

**Methods:**

Soft tissue paper mill workers at a Swedish paper mill were investigated using a questionnaire and lung function and atopy screening. Spirometry without bronchodilation was performed with a dry wedge spirometer, and forced vital capacity (FVC) and forced expiratory volume in 1 s (FEV_1_) were obtained and expressed as percent predicted. Exposure to soft paper dust was assessed from historical stationary and personal measurements of total dust, in addition to historical information about the work, department, and production. The impact of high exposure to soft paper dust (> 5 mg/m^3^) vs. lower exposure ≤ 5 mg/m^3^, as well as cumulative exposure, was analyzed using multiple linear regression models. Multivariate models were adjusted for smoking, atopy, gender, and body mass index.

**Results:**

One hundred ninety-eight current workers (124 male and 74 female) were included. There were significant associations between both cumulative exposure and years of high exposure to soft paper dust and impaired lung function. Each year of high exposure to soft paper dust was associated with a 0.87% decrease in FEV_1_ [95% confidence interval (CI) − 1.39 to − 0.35] and decreased FVC (− 0.54%, 95% CI − 1.00 to − 0.08) compared to the lower exposed workers.

**Conclusions:**

The present study shows that occupational exposure to soft paper dust (years exceeding 5 mg/m^3^ total dust) is associated with lung function impairment and increased prevalence of obstructive lung function impairment.

## Introduction

Occupational exposure to dust, both organic and inorganic, is clearly associated with lung function impairments and clinical outcomes like chronic obstructive pulmonary disease (COPD) and interstitial lung disease (Blanc et al. 2019). The pulp and paper industry is an important industrial sector in Sweden, and a large sector is the production of soft paper (FAO 2017). Growth in demand for particularly soft paper, i.e., toilet paper, paper towels, and napkins has been particularly strong in Asia (CEPI 2017). Soft paper mills still have high exposure to dust, and in previous decades, dust levels have frequently exceeded 10 mg/m^3^. Soft paper dust is an organic dust with a varying proportion of inorganic material depending on the use of additives (Sahle et al. 1990). In animal models, it has been shown that fibers from cellulose are biopersistent, and it has also been shown that exposure to cellulose dust are associated with fibrotic and granulomatous reactions (Muhle et al. 1997, Tatrai et al. 1996). Hence, it seems reasonable exposure to soft paper dust should be associated with impaired lung function.

We have in previous studies shown that high occupational exposure (> 5 mg/m^3^) to soft paper dust is associated with impaired lung function, mainly decreased forced expiratory volume in 1 s (FEV_1_) and forced vital capacity (FVC) (Ericsson et al. 1988; Järvholm et al. 1988). A German study, also on soft paper mill workers with occupational exposure (> 5 mg/m^3^) to soft paper dust, observed a dose–response relationship for cumulative exposure to soft paper dust and decreased FVC (Kraus et al. 2004). By contrast, in two studies with lower exposure levels (≤ 5 mg/m^3^), there was no association between exposure to soft paper dust and lung function impairment (Heederik et al. 1987; Thorén et al. 1989b). There is also conflicting data about whether exposure to paper dust increases the risk for asthma and COPD (Thorén et al. 1989a; Torén et al. 1991, 1994, 1996). Hence, there is an obvious need for further studies investigating the relation between exposure to soft tissue paper dust and respiratory health effects, especially lung function outcomes.

In this study, we have examined workers in a large soft tissue paper mill in Sweden with the aim to elucidate the extent to which exposure to soft paper dust is associated with respiratory health effects.

## Materials and methods

The study was performed at a mill where soft tissue paper production started on a small scale in 1948, and increased considerably around 1960. Today, the mill is one of the largest soft paper plants in Sweden. In 2006, all employees currently working at the mill (*n* = 205) were invited to participate in a clinical investigation at the mill site. Six of the invited persons did not participate. Hence, the initial study population included 199 workers.

All invited workers received an extensive questionnaire with questions about occupational history, smoking habits, and respiratory symptoms and asthma. Height and weight were measured with workers wearing light clothing and no shoes. Spirometry without bronchodilation was performed with a dry wedge spirometer (Vitalograph, Buckingham, UK) and according to American Thoracic Society (ATS)/European Respiratory Society (ERS) standards (Miller et al. 2005). Forced vital capacity and FEV_1_ were measured with individuals in a sitting position and wearing a nose clip, and predicted normal values were based on the GLI-equations (Quanjer et al. 2012). Blood samples were analyzed for specific immunoglobulin E class using Phadiatop analysis (Pharmacia & Upjohn Diagnostics, Uppsala, Sweden).

### Definitions

Different definitions of airflow limitation (AL) were used: AL_GOLD_ was defined, according to Global Initiative for Obstructive Lung Disease (GOLD) criteria, as an FEV_1_/FVC ratio of < 0.7 (Vogelmeier et al. 2017); AL_LLN_ was defined as an FEV_1_/FVC ratio below the lower limit of normal (LLN_5_) (Quanjer et al. 2012). Restrictive spirometric pattern was defined as FEV_1_/FVC > 0.7 and FVC < 80% predicted (Crapo 1994).

Smoking was classified as never-smoking, former smoking, and current smoking, based on the subjects’ answers to the questionnaire. Pack-years were calculated among current and former smokers. Asthma was defined as an affirmative answer to “Have you ever had asthma diagnosed by a physician?” and onset after 15 years of age (Torén et al. 1993). Cough with phlegm (chronic bronchitis) was defined as an affirmative answer to “Have you had long-standing cough with phlegm?” and “If so, did any period last at least 3 months?” and “If so, have you had such periods at least 2 years in a row?” (Holm et al. 2014). Wheezing was defined as an affirmative answer to the question “Have you experienced wheeze or whistling in your chest at any time since 15 years of age?” Atopy was defined as a positive Phadiatop result (class 1) (Matricardi et al. 1990). Body mass index (BMI) was defined as measured weight/height^2^.

### Exposure assessments

For the purpose of this study, we developed a specific job exposure matrix (JEM) for soft paper dust exposure. Exposure to soft paper dust was assessed from historical stationary and personal measurements of total dust, in addition to historical information about the work, department where worked, and kind of production, allowing us to assess exposure to soft paper dust for every year for each worker with an estimated mean level of dust (mg/m^3^). Further, the cumulative exposure, in mg/m^3^-years, was calculated for each worker, as (mg/m^3^) × years of exposure. Due to variations in exposure across time and duties, most workers were classified into more than one exposure category over the study period. The cumulative number of years in different exposure categories is shown in Table 1. Cumulative mg/m^3^-years for all workers were divided into quartiles and workers in the highest quartile (> 72 mg/m^3^-years) were defined as high exposed. The remaining workers were classified as lower exposed. High exposed years were defined as years having been exposed to soft paper dust exceeding 5 mg/m^3^.

**Table 1:** Categories of exposure to soft tissue paper dust by cumulative number of years of exposure working at the mill

Exposure category, total dust	Number of workers	Range of years worked (individuals)	Total of years worked (whole study population)
0–2 mg/m^3^	198	1–48	4283
1–5 mg/m^3^	109	0–26	680
5–10 mg/m^3^	51	0–11	263
> 10 mg/m^3^	26	0–12	131

### Statistical analyses

In the univariate analyses, we dichotomized the subjects into high exposed and lower exposed to soft paper dust. Univariate inferential analyses were performed using Chi-square test and Student’s *t* test. Where there were fewer than ten subjects in any stratum, Fisher’s exact test was used for univariate analyses. Univariate analysis results were considered significant if *p* < 0.05.

Lung function outcomes (dependent variable) and the association between the different independent variables (gender, BMI, pack-years, current smoking, atopy, and soft paper dust exposure) were examined in multiple linear regression models, and also stratified into never-smoking and ever-smoking. The associations between high exposure (highest quartile of cumulative dust exposure) and AL_GOLD_, AL_LLN_, asthma, chronic bronchitis, and wheezing were analyzed using logistic regression models. Dust exposure was measured in terms of high exposed years as well as the cumulative exposure measure, mg/m^3^-years. All variables were kept in the models even if most of them were without formal statistical significance. The models were adjusted for former and current smoking and also stratified into never-smoking and ever-smoking. In all regression models, we used 95% confidence intervals (CIs) and *p* values to determine significance. All analyses were performed using SAS version 9.4 (SAS, Cary, NC, USA).

## Results

One person was excluded due to inadequate spirometry technique; hence, the final study population comprised 198 workers with complete data regarding lung function and smoking habits. Basic data of the study population are shown in Table 2. In the univariate analyses, FEV_1_ was significantly lower among the high exposed compared to the lower exposed workers, 91.4% vs. 97.8% predicted. Further, the prevalence of both AL_GOLD_ and AL_LLN_ was higher (p < 0.05) among the high exposed workers, 27.5% vs. 6.8%, and 19.6% vs. 6.1%, respectively.

**Table 2:** Personal data age, gender, employment time, as well as data on respiratory health, smoking habits, pulmonary function, and dust exposure data in soft tissue paper mill workers

	All, *N* = 198	Lower exposed workers, *N* = 147	High exposed workers, *N* = 51	*p* value^a^
Mean age, years (SD)	48.2 (10.3)	45.6 (10.4)	56.6 (5.1)	< 0.001
Female workers	37.4% (*n* = 74)	34.7% (*n* = 51)	45.1% (*n* = 23)	0.19
Employment time, years (SD)	26.8 (11.5)	23.2 (11.0)	36.9 (5.5)	< 0.001
Mean cumulative exposure mg/m^3^-years (SD)	51.1 (50.0)	27.3 (19.9)	119.9 (46.9)	< 0.001
Never-smokers	39.9% (*n* = 79)	44.2% (*n* = 65)	27.5% (*n* = 14)	0.04
Ex-smokers	35.9% (*n* = 71)	34.0% (*n* = 50)	41.2% (*n* = 21)	0.36
Current smokers	24.2% (*n* = 48)	21.8% (*n* = 32)	31.4% (*n* = 16)	0.17
Pack-years among ever-smokers, mean (SD)	17.7 (11.9)	15.3 (11.3)	23.1 (11.4)	< 0.001
Atopy	22.2% (*n* = 44)	24.5% (*n* = 36)	15.7% (*n* = 8)	0.19
Body mass index, mean (SD)	26.6 (4.7)	26.4 (4.3)	27.2 (5.8)	0.28
Asthma	3.0% (*n* = 6)	2.7% (*n* = 4)	3.9% (*n* = 2)	0.65^b^
Wheezing	18.7% (*n* = 37)	19.7% (*n* = 29)	15.7% (*n* = 8)	0.52
Chronic bronchitis	4.0% (*n* = 8)	4.1% (*n* = 6)	3.9% (*n* = 2)	1.00^b^
FEV_1_ (% predicted) (SD)	96.1 (13.7)	97.8 (11.4)	91.4 (18.0)	0.004
FVC (% predicted) (SD)	99.5 (11.9)	100.4 (10.4)	96.8 (15.2)	0.07
AL_GOLD_	12.1% (*n* = 24)	6.8% (*n* = 10)	27.5% (*n* = 14)	0.003^b^
AL_LLN_	9.6% (*n* = 19)	6.1% (*n* = 9)	19.6% (*n* = 10)	0.01^b^
Restrictive spirometric pattern	1.5% (*n* = 3)	1.4% (*n* = 2)	2.0% (*n* = 1)	1.00^b^

*AL* airflow limitation, *AL*_*GOLD*_ AL according to Global Initiative for Obstructive Lung Disease criteria, *AL*_*LLN*_ AL with an FEV_1_/FVC ratio below the lower limit of normal, *FEV*_*1*_ forced expiratory volume in 1 s, *FVC* forced vital capacity, *SD* standard deviation

^a^High vs. low exposed

^b^Fisher’s exact test

In adjusted multiple linear regression models, lung function decreased for every high exposed year (Table 3). For each year of exposure to high levels of soft paper dust, there was a 0.87% predicted decrease in FEV_1_ (95% CI − 1.39 to − 0.35). A similar, but lesser, effect was seen for FVC (− 0.54% predicted, 95% CI − 1.00 to − 0.08). Among never-smokers, the exposure effect was significant only with regard to FVC (− 1.33% predicted, 95% CI − 2.50 to − 0.16) (Table 3). Cumulative exposure to soft paper dust expressed as mg/m^3^-years was associated with decreased FEV_1_ and decreased FVC (Table 3). This was seen among all workers and among ever-smokers. Among never-smokers the estimates also indicated decreased FEV_1_ and FVC, but without formal statistical significance.

**Table 3:** Multivariate linear regression analyses of lung function, in percentage of predicted values, among subjects currently employed (*n* = 198) at a soft tissue paper mill in Sweden

	% of predicted FEV_1_			% of predicted FVC		
	Estimate	95% CI	*p* value	Estimate	95% CI	*p* value
All (*n* = 198), high exposed years^a^	− 0.87	− 1.39 to − 0.35	0.001	− 0.54	− 1.00 to − 0.08	0.02
Never-smokers (*n* = 79), high exposed years^a^	− 1.16	− 2.47 to 0.14	0.08	− 1.33	− 2.50 to − 0.16	0.03
Ever-smokers (*n* = 119), high exposed years^a^	− 0.75	− 1.34 to − 0.15	0.01	− 0.37	− 0.89 to 0.15	0.16
Cumulative exposure						
All (*n* = 198), mg/m^3^-years	− 0.05	− 0.10 to − 0.003	0.043	− 0.04	− 0.07 to − 0.004	0.03
Never-smokers (*n* = 79), mg/m^3^-years	− 0.08	− 0.18 to 0.01	0.08	− 0.06	− 0.16 to 0.007	0.07
Ever-smokers (*n* = 119), mg/m^3^-years	− 0.05	− 0.10 to − 0.003	0.04	− 0.03	− 0.07 to 0.01	0.16

All models are adjusted for gender, atopy, body mass index, current smoking and pack-years. The models for never-smokers does not include smoking variables

*CI* confidence interval, *FEV*_*1*_ forced expiratory volume in 1 s, *FVC* forced vital capacity

^a^High exposed years > 5 mg/m^3^ total dust vs. lower exposed years ≤ 5 mg/m^3^ total dust

In the logistic regression models, high exposure to soft paper dust was associated with an increased odds ratio (OR) both for AL_GOLD_ (OR 4.6, 95% CI 1.8–12.0) for AL_LLN_ (OR 3.4, 95% CI 1.2–9.3) (Table 4). There were no significant associations with asthma, wheezing, or chronic bronchitis (Table 4).

**Table 4:** Logistic regression models of adult-onset asthma, wheeze, chronic bronchitis, and lung function parameters among subjects (*n* = 198) currently employed at a soft tissue paper mill in Sweden

	All workers		Never-smokers		Ever-smokers	
	Cases, *n*	High exposed workers, OR (95% CI)	Cases, *n*	High exposed workers, OR (95% CI)	Cases, *n*	High exposed workers, OR (95% CI)
AL_GOLD_^a^	24	4.6 (1.8–12)	2	–	22	3.5 (1.3–9.3)
AL_LLN_^a^	19	3.4 (1.2–9.3)	3	11 (0.9–134)	16	2.6 (0.9–7.9)
Asthma^a^	6	1.3 (0.2–8.0)	0	–	6	1.4 (0.2–8.7)
Wheezing^a^	37	0.7 (0.3–1.6)	10	0.5 (0.6–4.5)	27	0.7 (0.3–1.9)
Chronic bronchitis^a^	8	0.8 (0.2–4.3)	1	–	7	1.0 (0.2–5.3)

High exposed workers, compared to lower exposed workers, adjusted for former and current smoking, and atopy among all workers, atopy among never-smokers and current smoking and atopy among ever-smokers

*CI* confidence interval, *OR* odds ratio, *AL* airflow limitation, *AL*_*GOLD*_ AL according to Global Initiative for Obstructive Lung Disease criteria, *AL*_*LLN*_ AL with an FEV_1_/FVC ratio below the lower limit of normal

^a^1 = yes; 0 = no

## Discussion

The main finding from this study is that high exposure to soft paper dust (> 5.0 mg/m^3^) was associated with decreased pulmonary function. Previous studies have indicated restrictive lung function impairment associated with paper dust exposure; by contrast, the results from this study indicated obstructive impairment, as FVC was less affected than FEV_1_, and the prevalence of AL was increased among high exposed workers.

Our previous study, showing a restrictive impairment of lung function, was conducted at paper mills with dust levels often exceeding 10 mg/m^3^; hence, probably higher than the exposure, present and past, at the mill in this study (Järvholm et al. 1988). In the present mill, the levels in the 1980s were between 5 and 10 mg/m^3^ total dust, but exposure levels were later reduced to around 1–2 mg/m^3^ (Thorén et al. 1989b). By analyzing the association between lung function and high exposed years, we consider both the intensity and the duration of exposure (De Vocht et al. 2015). Our findings in the present study indicate that working for at least 1 year at dust levels exceeding 5.0 mg/m^3^ is associated with significant lung function impairment. Such high exposure levels have not been present in the mill in the last two decades; hence, the affected workers have had quite a long exposure to soft paper dust. However, low exposed workers with a similar duration of exposure did not show any signs of lung function impairment. Among never-smokers FVC was significantly decreased, − 1.30% predicted but FEV_1_ was not significantly affected.

Chronic airflow limitation (CAL), is commonly defined as an FEV_1_/FVC ratio of < 0.7 (Vogelmeier et al. 2017). This has been seriously challenged, however, because the fixed ratio FEV_1_/FVC < 0.7 does not take into account the age-related changes in lung function. Thus, it has been argued that employing a definition based on FEV_1_/FVC < 0.7 leads to an overestimation of airflow limitation in the older population (Pellegrino et al. 2005). An alternative approach that has been proposed is to use the LLN as a cut-off. The LLN is calculated using the distribution in reference material; the use of LLN has been proposed by the ATS/ERS (Pellegrino et al. 2005). However, as we only had access to spirometry without bronchodilation, we analyzed AL as a proxy for CAL. However, we observed, as expected, that the prevalence of AL_GOLD_ was higher (12.1%) than the prevalence of AL_LLN_ (9.6%).

We decide to present both AL_GOLD_ and L_LLN_ as a joint American Thoracic Society/European Respiratory Society (ATS/ERS) statement called for investigations of comparisons between the fixed cut-off (FEV_1_/FVC < 0.7) and the LLN-based definition (FEV_1_/FVC < LLN) of airflow limitation in predicting adverse health outcomes (Celli et al. 2015). Our results also indicated that both definitions predicted an adverse outcome.

Whether soft paper dust exposure increases obstructive lung disease risk is unclear. Among soft tissue paper workers, we have previously described increased mortality due to obstructive lung disease as well as an insignificantly increased incidence rate of asthma (Thorén et al. 1989a; Torén et al. 1994). In addition, soft paper workers seem to have an increased prevalence of rhinitis and irritative symptoms of the upper airways, even those exposed to levels below 5 mg/m^3^ (Thorén et al. 1989b; Hellgren et al. 2001; Kraus et al. 2002, Holm et al. 2011). Among more highly exposed workers, increased prevalence of cough has been reported (Torén et al. 1994; Kraus et al. 2002). A suspected case of occupational asthma due to cellulose has also been described (Knight et al. 2018). Our results provide further evidence that high exposure to soft paper dust has irritating effects on the airways, impairs lung function and increases the risk for AL, regardless of how this is defined. In a longer perspective exposure to soft paper dust may also increase the risk for COPD (Järvholm 2000).

We also intended to define a group of workers with restrictive spirometric pattern, but the prevalence was too low to perform any meaningful analyses.

The present study has a number of methodological limitations that have to be considered. The main limitation is the cross-sectional design. This design implies that workers with long-standing respiratory ailments may have left the mill. We have previously shown that subjects with asthma or respiratory symptoms have an increased frequency of job change (Torén et al. 2009). This turnover of workers will cause an underestimation of the risk associated with paper dust exposure, due to healthy worker selection bias (Östlin 1989). The reference group in the present study was not unexposed; rather, they were low exposed workers, which may also have resulted in underestimation.

Our analysis adjusted for current smoking and cumulative dose of tobacco (pack-years). There is, however, a strong relation between decreased FEV_1_ and increased prevalence of AL and tobacco smoking, and, hence, residual confounding by smoking cannot be excluded.

Another major limitation is the lack of power. The study size was limited to the workforce in one mill, which limited the number of study subjects. Still, there were significant associations between exposure and the lung function parameters.

## Conclusions

The present study shows that occupational exposure to soft paper dust (years exceeding 5 mg/m^3^ total dust) is associated with lung function impairment and increased prevalence of obstructive lung function impairment.
